# Visual Gait Analysis Based on UE4

**DOI:** 10.3390/s23125463

**Published:** 2023-06-09

**Authors:** Ruzhang Liu, Luyin Liu, Guochao Ma, Shanshan Feng, Yuanhui Mu, Dexi Meng, Shuying Wang, Enlin Cai

**Affiliations:** The School of Electronic Information, Qingdao University, Qingdao 266071, China; 2021023780@qdu.edu.cn (R.L.); 2021023769@qdu.edu.cn (L.L.); 2020020634@qdu.edu.cn (G.M.); fengshanshan@qdu.edu.cn (S.F.); mouyuanhui@qdu.edu.cn (Y.M.); mengdexi@qdu.edu.cn (D.M.); sy_w@qdu.edu.cn (S.W.)

**Keywords:** virtual reality, UE4 engine, 3D gait analysis, visualization

## Abstract

With the development of artificial intelligence technology, virtual reality technology has been widely used in the medical and entertainment fields, as well as other fields. This study is supported by the 3D modeling platform in UE4 platform technology and designs a 3D pose model based on inertial sensors through blueprint language and C++ programming. It can vividly display changes in gait, as well as changes in angles and displacements of 12 parts such as the big and small legs and arms. It can be used to combine with the module of capturing motion which is based on inertial sensors to display the 3D posture of the human body in real-time and analyze the motion data. Each part of the model contains an independent coordinate system, which can analyze the angle and displacement changes of any part of the model. All joints of the model are interrelated, the motion data can be automatically calibrated and corrected, and errors measured by an inertial sensor can be compensated, so that each joint of the model will not separate from the whole model and there will not occur actions that against the human body’s structures, improving the accuracy of the data. The 3D pose model designed in this study can correct motion data in real time and display the human body’s motion posture, which has great application prospects in the field of gait analysis.

## 1. Introduction

Virtual reality technology is a new emerging technology in recent years through the super-high computing power of the computer to achieve the visualization of complex data and the creation of interactive scenes, and has completely exceeded the interactive mode of traditional human–computer interface. The concept of virtual reality refers to a whole simulated reality that is built with computer systems using digital formats [1]. With the development of science and technology, virtual reality technology has developed rapidly, and its content covers many fields [2], such as retail [3,4], education [5], tourism [6], health care [7], entertainment [8] and research [9,10]. Virtual technology has been widely used and, relying on the current virtual technology for high precision and high simulation needs, it can almost perfectly show the object state model, as well as the elements of the environment rendering; more importantly, its powerful interaction ability is unprecedented.

Three-dimensional gait analysis is used to collect and analyze the data on posture movement, the center of gravity fluctuation and joint bending. In the medical field and professional sports field, the application of human gait analysis is very extensive [11,12,13,14]. Teuf et al. described an inertial sensor (IMU) system that accurately measures the ROM of human gait and the special characteristics of the 3D kinematics of the lower limbs, and is suitable for patients after THA and healthy people [15]. However, they did not use the biomechanical model to show the human lower limb movements in real time. Figueiredo et al. proposed a wearable inertial sensor system for real-time detection of 3D angular velocity and 3D acceleration for up to six lower limbs and trunk segments, and sagittal joint angle for up to six lower limb joints [16], but they do not have a 3D model which was used to show the human gait. Tham et al. demonstrated that when measuring joint angles using inertial sensors, NARX can avoid the influence of magnetometers, accurately estimate 3D knee joint angles and measure 3D joint angles in the long term [17]. In the same year, a full-body wireless wearable motion-sensing system was reported by Lee et al. to study the motion of human lower limbs and arms, which can reconstruct simple 3D human body models in real-time using quaternion data measured by sensors [18]. However, their 3D mannequin is composed of simple stick structures, not a complete mannequin, and, to some extent, can only reflect part of the limb movement and cannot vividly show the human body movement. Xie et al. proposed GaitTracker, an IMU-based three-dimensional (3D) skeletal tracking system, which is able to accurately perform 3D skeletal tracking of the lower limbs for gait analysis [19]. Nevertheless, they built the 3D model to show the human body of lower limb movement, but there is no comprehensive analysis of the upper limb movements. In fact, most gait analysis research is about the movement of part of human limbs and has not been visualized, while there are few reports about the visual gait analysis of the whole human body movement.

In this study, a 3D pose model was created by using UE4 software Version 2.24. The model can be combined with the motion capture module based on inertial sensors to obtain the motion data of 12 parts of the human body and display the various postures of the human body in real time in the form of animation. Using virtual reality technology, the gait data of humans is obtained by gait analysis of the model, which makes the analysis process clearer and the operation simpler. The experimental results show that the 3D posture model can meet the requirements of gait analysis, and the real-time visual change of virtual reality presentation is the core function of the current intelligent requirements, which can be widely used in medical and sports fields of 3D gait analysis.

## 2. Thesis Research Content

Shanshan Chen et al. pointed out that gait analysis is usually performed by subjective and qualitative approaches in current clinical settings, although some severe gait disorders can be observed by the human eye without quantitative measures, subtle changes can go unnoticed, thus affecting disease staging, severity assessment, and subsequent treatment planning [20]. So, sometimes there may be a deviation in analyzing the cause of disease by observing the motion trajectory with the naked eye, which makes the doctor unable to see the internal focus of the patient. Meanwhile, 3D gait analysis is generally used in medicine for the objective evaluation of human walking and can be used to supplement standard clinical evaluation in identifying and understanding gait problems [21]. A primary advantage of 3D gait analysis is the ability to quantify joint and segment movement in all three planes throughout the gait cycle (stance and swing), and this information can help clinicians identify gait deviations that are difficult to recognize and, in many cases, impossible to appreciate through observation alone [22]. Therefore, through the signal import of inertial sensors combined with virtual reality, the patient’s motion posture is displayed in an animated form, thereby assisting the doctor to diagnose the patient’s condition. In this study, the human model is built and imported into the UE4 platform, and the action programming is carried out by using C++, blueprint language and its port technology, to display the 3D pose animation model and for the medical and sports fields of 3D gait analysis.

Based on the analysis of human gait, this paper studies the modeling of the human model and the use of blueprint language and animation logic processing for the model, which constructs the basic mesh skeleton with the ability of independent movement and makes the human model joint linkage in order to complete the action demonstration under the instruction. Finally, the gait algorithm is combined with the model so that the model can be combined with the motion capture module based on inertial sensors, and data transmission can be carried out to facilitate the real-time display of various postures of human motion.

## 3. Model Design

Unreal Engine is a popular game engine for creating high-fidelity video games and one of the best choices for virtual reality developments [23]. At present, the use of UE4 can be described as very extensive, and it has a far-reaching impact on games, film and television, medical treatment, sports and so on [24,25,26,27,28,29,30,31]. The real-time visualization capability of the UE4 engine is not available in traditional virtual technology. Compared with traditional modeling software, its advantage lies in real-time rendering, which enables designers to create in the WYSIWYG state. The UE4 platform supports the interaction between software and hardware of different types of virtual reality devices and can receive files in various formats as creative materials, which greatly improves the richness of operation.

UE4 can be programmed in blueprint or C++, which makes it suitable for almost any virtual reality simulation scenario. Blueprints, also known as visual blueprint scripts, work through node wires and are a visual programming language that requires compilation, providing an intuitive, node-based interface for creating new types of Actor and Level Script events. Blueprints are a special asset type that can create logic, set variable data in an intuitive and node-based way, plan to create custom characters, events, features, etc., and quickly complete Gameplay iterations. Blueprint can also inherit from C++ classes, defined variables in C++, call functions or implement events in C++. In its basic form, a blueprint is a visual script that you add to your game. It uses wires to connect nodes, events, functions, and variables together to create complex gameplay elements. Blueprints have the advantage of being intuitive and convenient, compiling quickly, and you can choose blueprints when creating some complex programming.

The blueprint interface may be understood as a function set in the UE4 platform, and one or more functions may be added to other blueprints, and the blueprints may have the functions of the interface after being assigned to the interface. The summary is that the blueprint interface needs to define a function, called an interface, so that the whole project exists this function for us to use, but in different blueprint classes, it can play different roles.

### 3.1. Skeleton Model

A skeleton mesh is a bone-bound model that you can use to create animation. In this study, a hierarchical skeleton model of the 3D human body is established, and the motion data of each node is corresponding to the 3D human body model for motion visualization. The building of the model requires a character skeleton, a mesh body, and a physical structure. Many of these modeling resources are included in the external resources owned by the UE4 platform, such as geometry, character body, etc. When adding skeletons and skins to the model, the resources of the model needed to be downloaded for use and put into the content folder. Then, open my character blueprint class, switch to the viewport window, select the Mesh object, choose to import skeleton in the details window on the right side, and adjust the position of the character model. With the skeleton in the create stage, the field of each limb can input all the values to define the joint setting of the role. Inside the skeleton settings panel can change the skeleton’s hierarchy or the number of joints. The character components are programmed by UE4 internal code to become a model with multiple blueprint-like functions, including skeleton construction, mesh construction, joint chain and so on.

### 3.2. Research on Model Coordinate System

When studying the motion of a model, the parameters such as motion angle and motion direction are inseparable from the coordinate system. The precise location of the nodes of the model is also inseparable from the coordinate system. The space coordinate systems mainly involved in attitude algorithms include the navigation coordinate system and the carrier coordinate system. A navigation coordinate system is a coordinate system used to transform and calculate the attitude change behavior of a moving carrier, and a carrier coordinate system generally refers to a follow-up coordinate system established on the carrier.

The model studied in this paper mainly uses the navigation coordinate system and the carrier coordinate system in each part of the model, as each part has its own independent coordinate system, according to its own coordinate system for XYZ axis rotation and displacement movement. Generally, the right-handed coordinate system is used for attitude algorithm, and because the coordinate system in UE4 is a left-handed coordinate system, in this case, the hardware-generated quaternions directly input into the model will lead to attitude mismatch. From the point of view of a vector product, the attitude can be consistent by adjusting the order and sign of the quaternion.

### 3.3. Data Transmission

In the general motion recording process, the motion data should be transmitted to software such as 3DMAX and adjust the animation effect in the motion capture software. Reorient the character’s skeletal structure and save the animation in FBX format to view. The main function of UE4’s live link is data transmission, which can directly import data into the engine and view the action effect. However, there is usually a big problem: the displacement information for the ROOT point is missing. The data from the external software is recorded by the hip as the model’s motion patterns change, and the bone structure corresponds to the marked points on the motion capture suit. During this process, the root skeleton, which marks the actor’s position information in project space, does not move because reality does not have these data.

In order to record the correct displacement information of the model animation, it is necessary to ensure that the root point of the actor can correctly capture the transmitted displacement data, and then drive the model bone animation and capture the same effect. In some cases, it is also necessary to correct the position through the collision of the character’s capsule body.

The hierarchy of character control in UE4 is capsule to root bone to hip pelvis. Therefore, pelvic data cannot be used in the other two levels. This effect was equivalent to using a low-level child object to drive a high-level parent object, and the movement of the high-level parent object would in turn drive the child object. The cycle would be endless, and the character would collapse in an instant. Thus, it needs to have the displacement and rotation values related to the low-level hip, and then import the high-level capsule body and root bone. Because the height of the pelvis is not equal to the height of the character, a new root point must be calculated at the same time to ensure that it is in the same position as the capsule body.

### 3.4. Model Joint Linkage

The human body model can be simplified to be composed of 19 segments and 20 joints. This paper mainly studies the movement angle and displacement changes of the 12 parts of the human body, including left and right feet, left and right calves, left and right thighs, waist, head, left and right upper arms and left and right small arms, and the movement data measured by Based on the mpu6050 inertial sensors can control the movement of the corresponding parts of the model.

**Figure 1:**
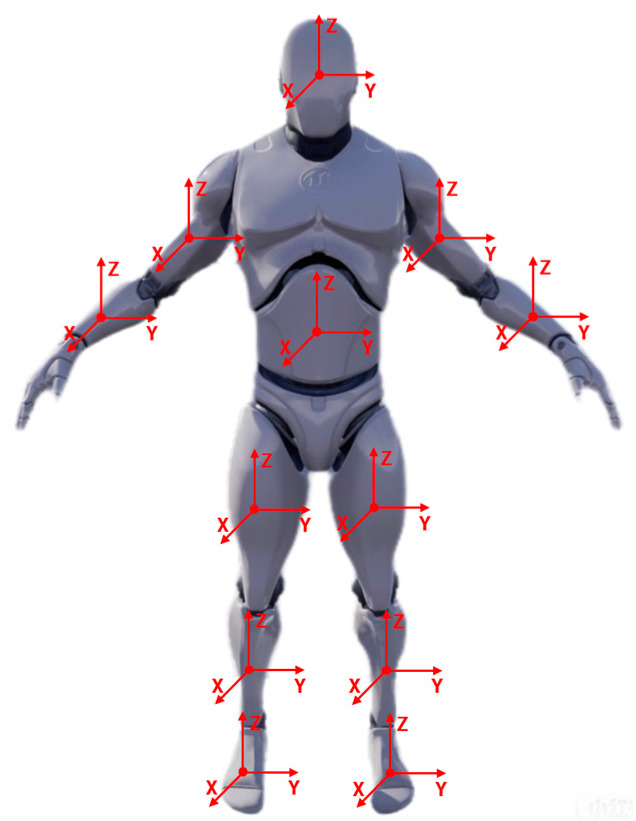
Human body model.

**Figure 2:**
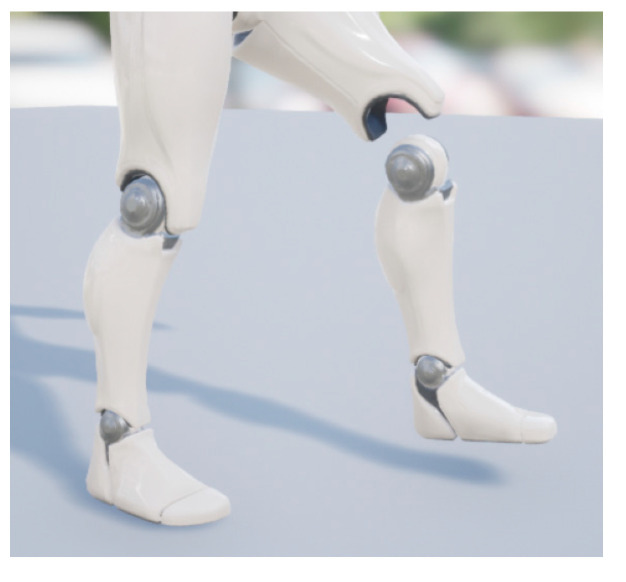
Model not linked.

As shown in Figure 1, each relevant part of the model has an independent coordinate system; that is, while the model is in motion, any part of the model will consist of three-axis gyroscope signals, three-axis acceleration signals and three-axis magnetometer signals. Therefore, the motion angle and displacement of each part of the model are calculated independently and then integrated into one piece to control the motion of the model. Due to various reasons, the motion data may have errors, and lead to possible separation of limbs. As shown in Figure 2, the left calf and left thigh of the model are separated. In order to avoid this situation, we use C++ and a blueprint language program to realize the ergonomic linkage of the model. With the human’s waist joint as the core, when a certain joint had to deviate from the whole model due to the measurement data of the inertial sensor, the model can calculate the offset of the joint in a certain direction and automatically minus a certain offset in the measurement data after to ensure it can reflect the human body movement posture so that the motion data for the calibration, correction and compensation part of the error of inertial sensor measurement. For example, the movement of the left thigh is associated with the movement of the left calf, and the influence of the movement of the left thigh must be considered when calculating the displacement of the left calf, so each joint of the model must be linked, and when an error in the movement data causes a certain part to be separated from the model, the model will correct the movement data to connect the limb parts, and the measurement accuracy is further improved. In the meantime, restrictions were also placed on some parts of the model to prevent unscientific human movements, such as rotating the head 180° horizontally.

### 3.5. Overall Introduction of the Model

**Figure 3:**
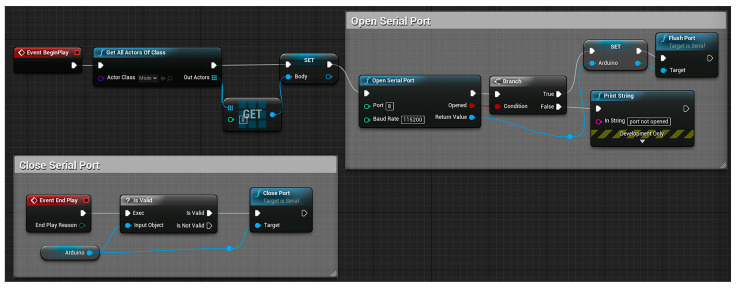
Blueprint for virtual serial port setup.

**Figure 4:**
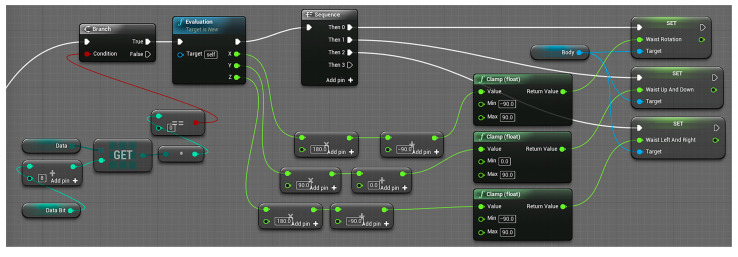
Accept lumbar joint data blueprint.

The three-dimensional posture model is used to combine with the motion capture module based on inertial sensors to display the motion posture of each part of the human body in real-time for gait analysis. Therefore, this study used blueprint language and C++ to program, created a virtual serial port, received the original data of motion in real-time through the serial port, and processed the data with a Kalman filter and complementary filter attitude algorithm to reduce certain errors. Finally, each set of quaternion data is transformed into Euler angles, which are applied to 12 parts of the model to display the motion posture in real time. Part of the code is shown in Figure 3 and Figure 4.

Figure 3 is a virtual serial port established by using blueprint language, which transmits motion data by changing the corresponding serial port number and baud rate. Figure 4 shows the blueprint for receiving lumbar joint data. The function of this code is to convert and apply the lumbar motion data transmitted through the virtual serial port to the model.

**Figure 5:**
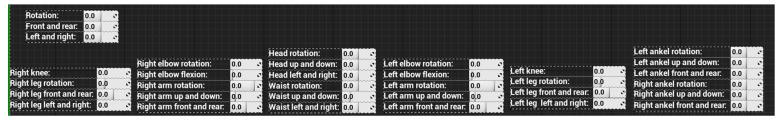
Joint angle display interface.

In this study, in order to better gait analysis, an interface for real-time display of each part of the human body movement angles was created, as shown in Figure 5. When the model receives the data movement, the angle of joint movement can be displayed on the interface in real time. In turn, you can control the animation of bones by inputting data.

**Figure 6:**
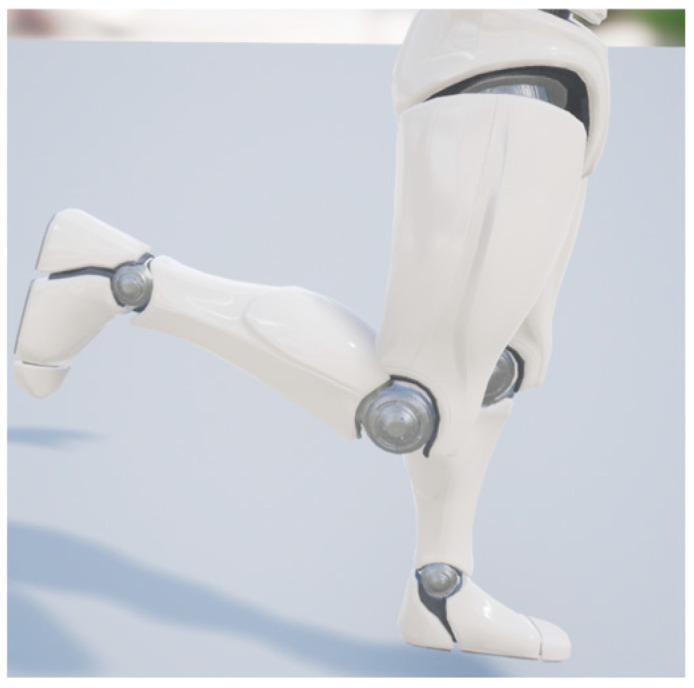
Joint angle automatically corrected to 90°.

**Figure 7:**
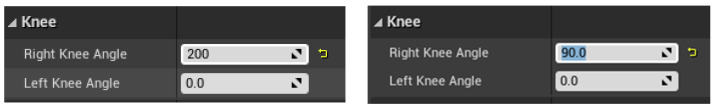
The system automatically corrects the maximum angle rear interface.

The three-dimensional posture model can receive the motion data and perform corresponding actions. The variables of the joint motion angle are set in the mode, and through the variables in the mode to communicate. The windows of 12 joints of the human body were set up in this study, and the sub-windows of each joint can input the corresponding angle of movement and rotation. Considering the actual situation of human motion, some restrictions are added to the corresponding joints, so that it cannot take unreasonable actions (such as rotating the foot by 180°). By using the Control Rig plug-in, when the data is greater than the bending rotation angle of a joint, it will automatically be set to the maximum variable angle. Examples are shown in Figure 6 and Figure 7 below.

As shown in Figure 6 and Figure 7, when you enter 200 (greater than the maximum angle that can be reached) in the right knee angle, the system automatically changed the parameter to 90, avoiding unscientific actions of the character.

**Figure 8:**
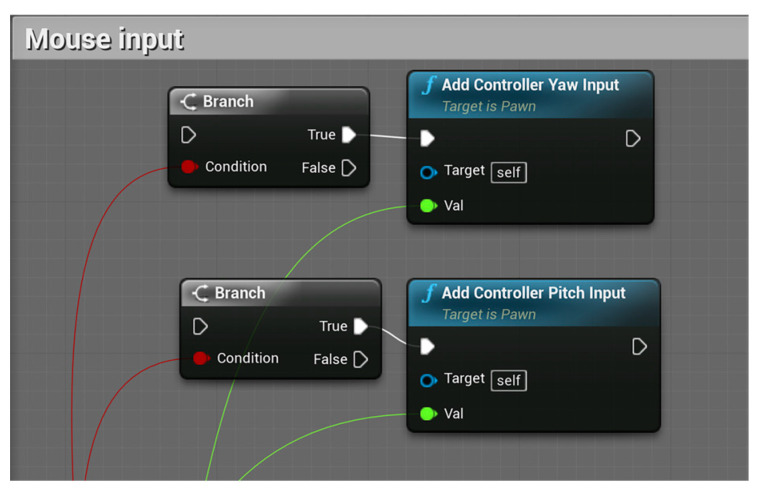
Mouse input blueprint.

In the operation of the model, sometimes in order to facilitate the debugging of different posture movements, it is very cumbersome to input data again and again, but it is more convenient to use the mouse to directly change the posture of the model. Therefore, the mouse input blueprint is added to use the mouse to move and rotate the limbs in real time. The function of this blueprint is to make the mouse control the joint to rotate and swing around the rotation axis. The blueprint is shown in Figure 8.

**Figure 9:**
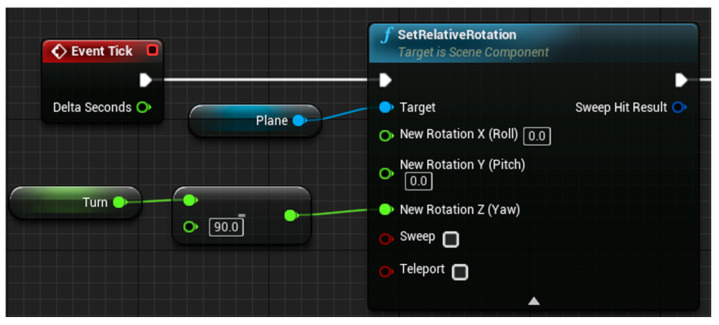
Event blueprint.

The custom event blueprint had an execution pin and an optional output data pin, which is equivalent to an initial switch of the model. When the event is set, it is equivalent to turning on the switch, which can call the event anywhere in the blueprint sequence, enabling the model to have the blueprint function of executing the pin. So, we set up the event blueprint, and then connected the blueprint for limb movement and rotation to the event blueprint node, as shown in Figure 9.

The blueprint of the whole model is divided into three parts: forward and backward movement, left and right movement and rotation. Therefore, the blueprint contains three groups of Delta Location X, Delta Location Y and Delta Location Z, and made the model body able to move in XYZ three directions and rotate. The movement blueprint of the limb is similar to the body movement blueprint, which includes the movement data of the XYZ three-axis directions and can realize the function of limb movement and rotation. These blueprints are assembled by connecting nodes, starting with event blueprints, and finally forming the control blueprint for the entire model.

**Figure 10:**
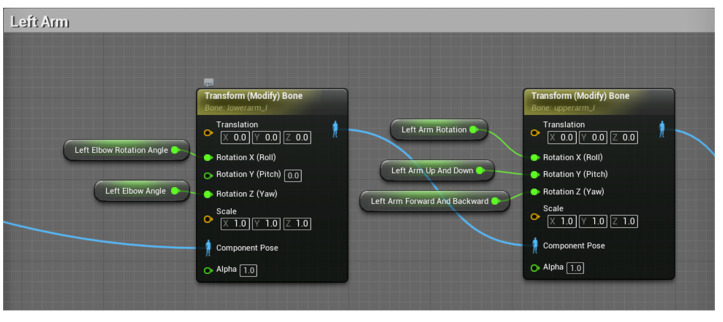
Left arm, left elbow exercise blueprint.

The blueprint of the left arm part is shown in Figure 10, driving the motion of the bones by applying the variables in mode to the bone model. The left arm blueprint is divided into two parts. One part controls the lateral elevation, forward and backward swing and pivot movement of the left upper arm, and the other part controls the movement of the left lower arm around the elbow joint in three directions with the left elbow as the node.

## 4. Experiments and Discussions

### 4.1. Partial Joint Experiment

**Figure 11:**
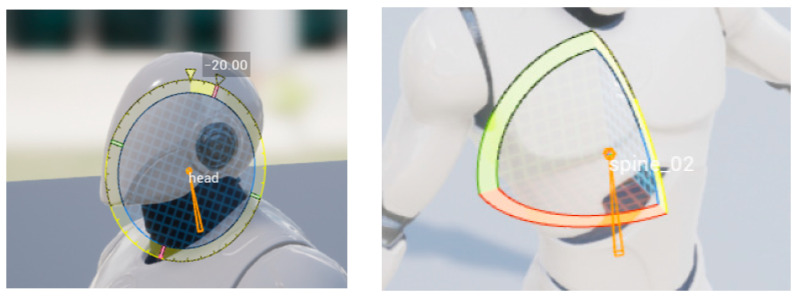
Head control and waist control.

The joint experiment of the model is related to its 12 parts, including the head, left and right arms, left and right elbows, left and right legs, left and right ankles, left and right knees, and waist. In order to make the model meet the action of three-dimensional gait analysis, the joint test is carried out first to test whether the joint can complete the corresponding action. Part of the joint input tests are shown in Figure 11. Through many experiments, it is concluded that each joint of the model can complete the action display according to the motion data.

### 4.2. Whole Model Experiment

**Figure 12:**
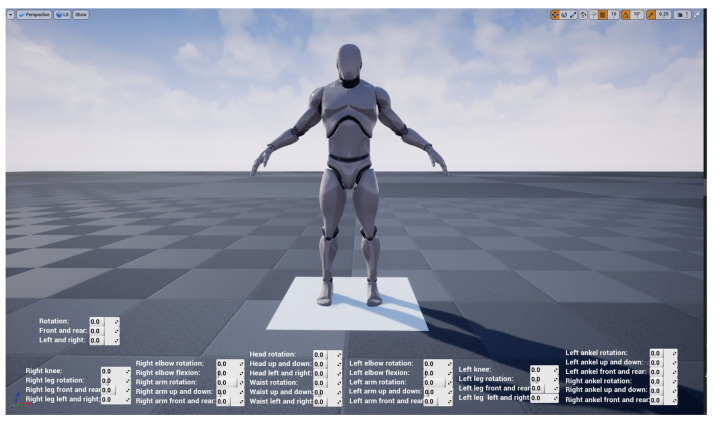
Whole model.

**Figure 13:**
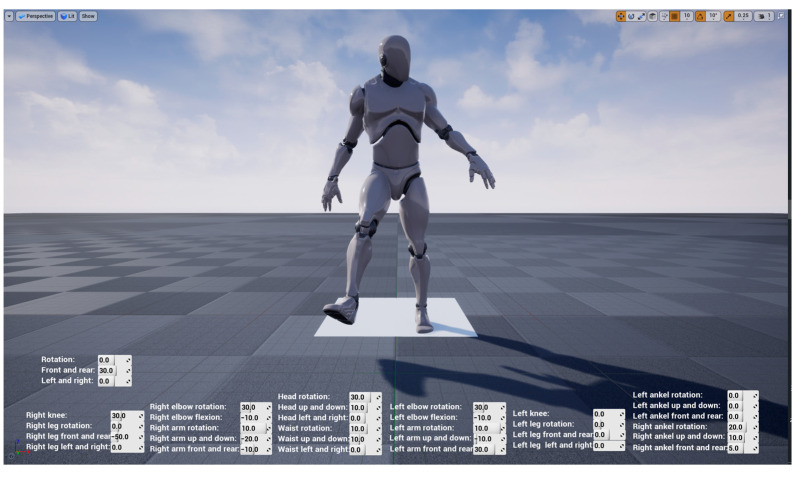
Whole model experiment.

The main interface during the model operation is shown in Figure 12. The character model consulted the small white man model inside UE4. Each joint of the model is independent but interrelated; that is, each part has a small coordinate system, which can independently control the movement of a certain part, and each joint is linked with other joints to ensure that it will not be separated from the whole model and ensure the reliability of data. The lower part of the main interface can display the change of each joint angle when the human body moves in real time. While there is no data transmission, the posture of the model can be controlled by changing the joint angle value in the main interface, as shown in Figure 13.

## 5. Conclusions

In this study, a 3D animation model for gait analysis is designed which can be combined with the motion capture module based on inertial sensors to vividly display the human motion posture through animation. The model can accept human motion data for gait calculation, and perform corresponding action demonstrations to achieve visual gait analysis. First of all, the model can receive the motion data measured by the motion capture module based on the inertial sensor through the virtual serial port, and perfectly present the motion of the signal source of the sensor and the angle of joint motion on the UE4 platform. At the same time, in order to avoid actions against the human body structure, all joints of the model are related to each other to achieve ergonomic linkage, so that the activities of the characters will not collapse while correcting part of the motion data to improve the accuracy of gait analysis.

During the experiments, the entire model was able to show the rotation and motion of 12 parts of the human body based on the motion data. At present, it is mainly used for gait measurement and analysis of low-speed main joints, but the details such as hand joints and toes have not been measured, and when the whole model moves at a high speed, the range of motion of each joint remains to be verified. All in all, this study improved the human three-dimensional posture testing system, which can visualize the human motion posture, and has a great application prospect in the field of medical rehabilitation and motion analysis.

## Data Availability

The study did not report any data.
